# Flipping the transcriptional switch from myelin inhibition to axon growth in the CNS

**DOI:** 10.3389/fnmol.2015.00034

**Published:** 2015-07-17

**Authors:** Jason B. Carmel, Wise Young, Ronald P. Hart

**Affiliations:** ^1^Brain Mind Research Institute and Departments of Pediatrics and Neurology, Weill Cornell Medical CollegeNew York, NY, USA; ^2^Burke-Cornell Medical Research InstituteWhite Plains, NY, USA; ^3^W.M. Keck Center for Collaborative Neuroscience and the Department of Cell Biology and Neuroscience, Rutgers UniversityPiscataway, NJ, USA

**Keywords:** DRG, mRNA expression, axon growth, IL-6, metallothionein, SLPI

## Abstract

Poor regeneration of severed axons in the central nervous system (CNS) limits functional recovery. Regeneration failure involves interplay of inhibitory environmental elements and the growth state of the neuron. To find internal changes in gene expression that might overcome inhibitory environmental cues, we compared several paradigms that allow growth in the inhibitory environment. Conditions that allow axon growth by axotomized and cultured dorsal root ganglion (DRG) neurons on CNS myelin include immaturity (the first few postnatal days), high levels of cyclic adenosine mono phosphate (cAMP), and conditioning with a peripheral nerve lesion before explant. This shift from inhibition to growth depends on transcription. Seeking to understand the transcriptome changes that allow axon growth in the CNS, we collaborated with the Marie Filbin laboratory to identify several mRNAs that are functionally relevant, as determined by gain- and loss-of-function studies. In this Perspective, we review evidence from these experiments and discuss the merits of comparing multiple regenerative paradigms to identify a core transcriptional program for CNS axon regeneration.

## Regenerative Paradigms to Identify Genes

To boost axon growth in the Central Nervous System (CNS), one strategy is to alter the CNS environment, to make it more conducive to growth by eliminating growth inhibitors or by adding a growth substrate, such as cells or material scaffolds. The other general approach is to increase the regenerative potential of neurons so that they may be able to grow in spite of the inhibitory environment. We considered the interplay of these two factors by asking which changes in gene expression could allow axonal regrowth in the inhibitory CNS environment. This Perspective is intended to review our approach and is not intended as a comprehensive review of regeneration-associated genes, which has been done by others, including in this issue (Ma and Willis).

One of the most actively investigated paradigms of regeneration is the conditioning lesion model. Axotomy of the peripheral dorsal root ganglion (DRG) axon normally results in regeneration while injury of the central axon does not. If the peripheral branch is injured first, however, and the central axon is injured 1 day to 2 weeks afterwards, the central axons regenerate to a much greater extent (e.g., McQuarrie and Grafstein, 1973; Richardson and Issa, 1984; Chong et al., 1999; Neumann and Woolf, 1999). The peripheral conditioning lesion induces changes in the axonal growth capacity of the injured neuron. The change in axonal growth state is likely due to induction of gene products associated with regenerative function, often called regeneration-associated genes or RAGs (Lankford et al., 1998). Smith and Skene (1997) demonstrated that cultured DRGs, which normally extend short, highly branched axons, will extend long, sparsely branched axons after a peripheral lesion. The switch from “arborizing” to “elongating” growth requires transcription and is blocked by addition of the transcription factor IIH (TFIIH)-associated protein kinase inhibitor 5,6-Dichloro-1-β-D-ribofuronosylbenzimidozole (DRB; Yankulov et al., 1995; Smith and Skene, 1997).

The conditioning lesion paradigm was used as an early strategy to screen for candidate gene products associated with re-growth. A prototypical RAG is GAP43. GAP43 was originally identified in a two-dimensional protein electrophoresis comparing DRGs with and without sciatic nerve injury (Skene and Willard, 1981). GAP43 is primarily localized to the growth cone and is elevated in other growth states, such as during development (Skene, 1989; Benowitz and Routtenberg, 1997), and recovery of function after injury is correlated with induction of GAP43 (Skene and Willard, 1981; Skene, 1989; Gispen et al., 1991; Plunet et al., 2002). Furthermore, overexpression of GAP43 and cytoskeleton-associated protein-23 (CAP23, encoded by the LOC10910172 gene in rat) increased the regeneration of dorsal column neurons after spinal cord injury (Bomze et al., 2001).

Based on this early success, several groups screened neurons after peripheral nerve lesion to isolate additional, putative RAGs. Techniques to isolate these changes include differential display (Kiryu et al., 1995; Su et al., 1997; Kim et al., 2001; Schmitt et al., 2003), expressed-sequence-tag (Tanabe et al., 2000), subtractive hybridization (Mladinic et al., 2005), and microarray (Fan et al., 2001; Bonilla et al., 2002; Costigan et al., 2002). Modeling the utility of microarrays for gene discovery, Bonilla and colleagues compared gene expression of mouse DRGs with and without nerve injury and found several genes whose expression differed between the groups (Bonilla et al., 2002). This group then showed that one of the gene products, small proline-rich repeat protein 1A (SPRR1A), when overexpressed, co-localized with actin in the growth cone and augmented axonal growth, even on inhibitory substrates. Reduction of SPRR1A restricted neurite outgrowth *in vitro*. This work highlights the utility of this approach for finding novel RAGs.

Another paradigm used to explore CNS regeneration is development. While adult mammalian CNS axons demonstrate little to no regeneration after injury, axons of young neurons may regrow (Bates and Stelzner, 1993; Hasan et al., 1993). A key event in the developmental loss of regenerative capacity is the postnatal myelination of long tract fibers. Evidence for this hypothesis includes experiments showing that delaying onset of myelination through immunological means extends the permissive period for regeneration (Keirstead et al., 1992, 1997). However, the loss of regenerative potential is due not only to the presence of myelin but also to the responsiveness of neurons to myelin. In particular, many types of neurons change their response to the myelin component myelin-associated glycoprotein (MAG), switching from growth promotion to inhibition during development (McKerracher et al., 1994; Mukhopadhyay et al., 1994; DeBellard et al., 1996). In DRGs this switch from promotion to inhibition occurs in a short period of time at postnatal day 3–4 (Johnson et al., 1989; Mukhopadhyay et al., 1994; DeBellard et al., 1996). A similar rapid decline in growth is seen in DRGs plated on myelin, but not those plated on the permissive substrate polylysine, at P2–3 (Cai et al., 2001).

Filbin and colleagues have shown that cyclic adenosine mono phosphate (cAMP) levels increase with conditioning lesion (Qiu et al., 2002) and decrease during development (Cai et al., 2001), paralleling regenerative potential. Treatment with cAMP increases the ability of older neurons to grow on myelin (Cai et al., 2001). The strength of the *in vitro* effects are similar to that of a conditioning lesion (Qiu et al., 2002), and intraganglionic administration of cAMP can mimic the effect of the conditioning lesion on dorsal column axon growth (Neumann et al., 2002; Qiu et al., 2002). Administration of the protein kinase A (PKA) inhibitor H89 blocks the growth of previously lesioned neurons (Qiu et al., 2002) or postnatal day 1 (P1) neurons on myelin, and the PKA inhibitor KT5720 decreases the number P2–3 corticospinal tract axons that grow into an embryonic tissue graft (Cai et al., 2001). The Filbin lab also showed that the increased growth after administration of cAMP depends on transcription, and they implicate the gene arginase-1 as an indispensable RAG in this system (Cai et al., 2002). It is not known whether exogenous cAMP completely recapitulates the regenerative capacity of DRG neurons early in development or following conditioning lesion, so we investigated all three methods to find genes regulated in common in all three models.

Thus, these studies probed three robust paradigms for CNS regeneration: young developmental stage, conditioning lesion and cAMP administration. All depend on cAMP signaling (as evidenced by blocking the effect with PKA inhibition), and both conditioning lesion and direct application of cAMP require transcription to activate outgrowth. All three paradigms are carried out in rat DRGs, cells that survive axotomy and can be easily cultured (Coggeshall et al., 1997). We examined gene expression differences between neurons with high growth capacity and those with low capacity to grow in a CNS environment. We hypothesized that gene expression differences that are in common between each of these paradigms would represent common and important RAGs.

Genes associated with regeneration may function by changing their expression levels either up or down. However, most previously-defined RAGs have increased levels in high growth states (e.g., GAP43, SPRR1A, and tubulin isoforms). The approach we took to isolate common RAGs, therefore, was one comparing the genes that were increased with cAMP treatment and conditioning lesion and decreased during development. These changes correspond to the changes in cAMP levels noted by the Filbin group in each of these paradigms (Cai et al., 2001; Qiu et al., 2002). Therefore, we were most interested in the subsets of genes with increased expression in the cAMP and conditioning lesion paradigms or decreased expression during development. Results identified a large number of genes (223) that were altered in the predicted ways by one or more of the regeneration paradigms. We were surprised, however, that there was little overlap in the candidate RAGs (7 total). This suggests the different paradigms that allow axon growth in the CNS environment may achieve regeneration through parallel mechanisms.

## Candidate Regeneration-Associated Genes

**Figure 1:**
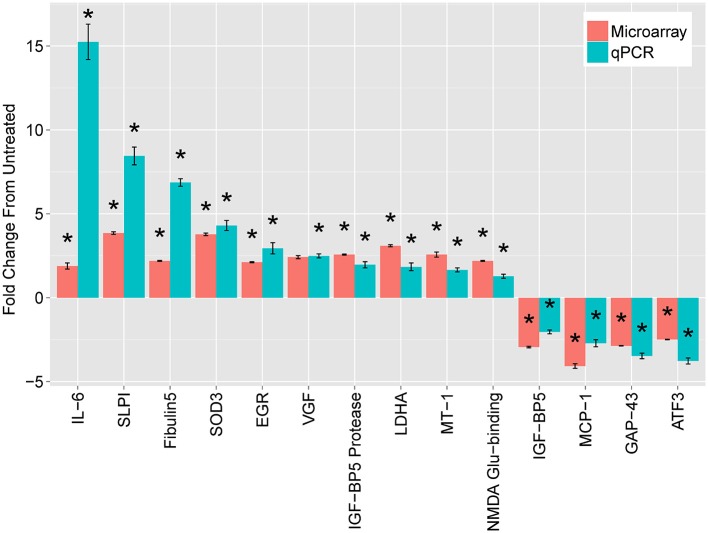
**Leading mRNA changes at 18 h following dbcAMP treatment.** The top mRNAs identified by microarray (red bars), compared with quantitative real-time PCR (qPCR, blue bars). Cultured, dissociated cells from L4 and L5 rat DRGs were treated with or without 1.5 mM dbcAMP for 18 h, harvested, and used to extract total cellular RNA. For microarrays, treated samples were labeled with one dye (Cy5) and untreated samples with another (Cy3), which were mixed and hybridized to a spotted-oligo, glass-slide array (Carmel et al., 2004). The dynamic range of the two-color microarray technique is reduced compared with qPCR and more recent technologies such as RNA-seq. Results are mean fold-change ± SEM, *n* = 3, *p < 0.05 Student’s *t*-test.

To validate the candidate genes, we first focused on comparison of DRGs treated with cAMP (at 18 h) with untreated DRGs. We targeted the validation on genes common to the three regeneration paradigms. We also included a few genes whose expression was strikingly divergent between the paradigms. We compared changes in gene expression by DRGs with and without exposure to cAMP for 18 h using both microarrays and quantitative Polymerase Chain Reaction (qPCR), which has a greater dynamic range. Microarray design and methods were described previously (Carmel et al., 2004). Selected results are shown in Figure 1. The full results of the microarrays can be found at NIH GEO with accession numbers GSE69466 and GSE69467.

The most striking finding was a large increase in Interleukin-6 (IL-6) expression. At 18 h, IL-6 increased 15-fold in DRG neurons treated with cAMP compared with untreated DRG, as measured by qPCR. In addition, the levels of IL-6 mRNA dramatically increased (77-fold) two days after lesion compared to those without injury. While IL-6 had the greatest change of any transcript for both cAMP treatment and conditioning lesion, IL-6 mRNA levels did not change significantly during the first five postnatal days. Due to the magnitude of the gene expression changes with cAMP and conditioning lesion, we pursued IL-6 as a possible RAG.

**Figure 2:**
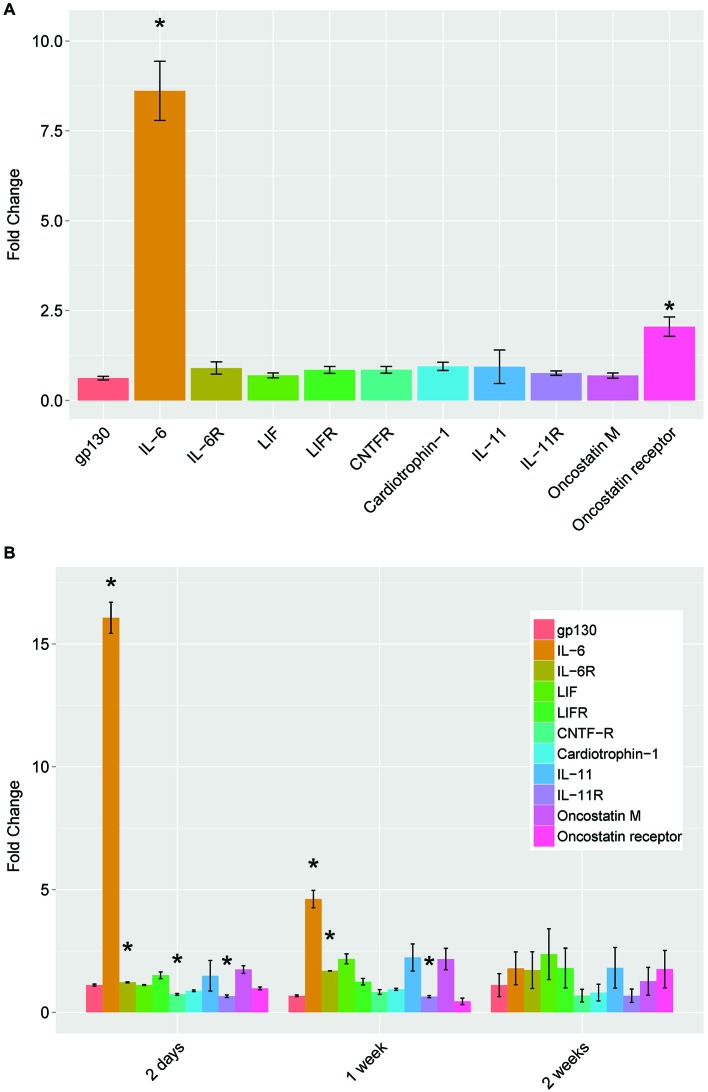
**mRNA levels of IL-6 family neuropoietic cytokines and their receptors by qPCR. (A)** dbcAMP treatment for 18 h. Dorsal root ganglion (DRG) cultures were prepared and treated as described in Figure 1 and total cellular RNA was prepared and assayed by qPCR. **(B)** Conditioning lesion at three time points. The sciatic nerve was surgically transected mid-thigh at postnatal day 20–24. After the times indicated, L4 and L5 DRG were removed and processed for total cellular RNA and assayed by qPCR. Results are mean fold-change SEM, *n* = 3, *p < 0.05 Student’s *t*-test.

Initially described for its role in inflammation and other immune functions, IL-6 is part of the neuropoietic family of cytokines. This family includes ciliary neurotrophic factor (CNTF), leukemia inhibitory factor (LIF), oncostatin M, cardiotrophin-1, and interleukins 6 and 11. These molecules bind to specific cytokine type 1 receptors, but all members require the common receptor gp130 for signal transduction. To determine if the changes in IL-6 with cAMP and conditioning lesion were limited to IL-6 or may involve other members of the gp130 family, we assayed the levels of mRNA for the cytokines and their receptors (Figure 2). For both 18 h of cAMP treatment (Figure 2A), and for the conditioning lesion (shown at several time points in Figure 2B), the predominant change is in IL-6, with more modest effects in other gp130 family members.

Subsequent experiments carried out at the Filbin laboratory demonstrated that IL-6 is a functional RAG (Cao et al., 2006). In addition to mRNA induction, IL-6 protein is also induced in DRGs and hippocampal neurons grown on inhibitory substrates and treated with cAMP. Administration of IL-6 protein mimics the effect of cAMP, reversing the growth inhibition of both DRGs and hippocampal neurons grown on inhibitory substrates. Importantly, exogenous IL-6 did not cause increased neurite outgrowth of DRGs grown on the permissive substrate poly-L-lysine. This suggests that IL-6 specifically reverses inhibition and is not due to a general trophic effect. Importantly, intrathecal delivery of IL-6 to DRG neurons blocks inhibition by myelin when they are explanted *in vitro* and *in vivo*, effectively mimicking the conditioning lesion.

However, the effects of cAMP and the conditioning lesion did not depend on IL-6. Blocking IL-6 signaling did not affect the ability of cAMP to overcome myelin inhibitors. In addition, IL-6-deficient mice respond to a conditioning lesion as effectively as wild-type (wt) mice. These data suggest that IL-6 can mimic both the cAMP effect and the conditioning lesion effect but is not an essential component of either response. IL-6 has also been found to promote the expression of other RAGs and to promote neurite outgrowth of cortical neurons (Yang et al., 2012). That IL-6 is sufficient but not necessary for axon regeneration fits with a larger point from these studies: the regeneration paradigms seem to act through parallel gene expression programs each of which is effective but not reliant on the others.

The second largest change in gene expression after cAMP administration was in secretory leukocyte protease inhibitor (SLPI), which was also identified in a screen of gene expression changes after spinal cord injury (Urso et al., 2007). SLPI is a serine protease inhibitor belonging to the family of whey acidic protein motif-containing proteins (Thompson and Ohlsson, 1986; Eisenberg et al., 1990). Gain- and loss-of-function studies in the Filbin lab show an essential role of SLPI in axon regeneration (Hannila et al., 2013). SLPI can overcome inhibition by CNS myelin and significantly enhance regeneration of transected retinal ganglion cell axons in rats. Furthermore, regeneration of dorsal column axons does not occur after a conditioning lesion in SLPI null mutant mice, indicating that expression of SLPI is required for the conditioning lesion effect. Thus, SLPI is both sufficient and necessary for axon regeneration in the damaged CNS.

The final putative RAG tested by the Filbin lab is metallothionein (MT). MTs are small cysteine-rich, zinc-binding proteins expressed throughout the CNS. Two closely related isoforms, MT-I and MT-II, when administered together, promote neurite outgrowth in adult DRG’s in the presence of myelin inhibitors (Siddiq et al., 2015). Likewise, a single intravitreal injection of MT-I/II after optic nerve crush promotes axonal regeneration. In contrast, adult DRGs from MT-I/II-deficient mice extend significantly shorter processes on MAG compared to wt DRG neurons, and regeneration of dorsal column axons does not occur after a conditioning lesion in MT-I/II-deficient mice. These experiments suggest that MT, like SLPI, is both necessary and sufficient for axon regeneration in the CNS.

## Additional Candidate Genes

Several additional transcripts were identified but we have not yet pursued their roles in regeneration. For example, VGF encodes a secretory-peptide precursor involved in plasticity and metabolism. VGF is modulated *in vivo* by paradigms which lead to neurotrophin induction, synaptic remodeling and axonal sprouting (Snyder et al., 1998a). VGF shows greatest expression during times of axonal outgrowth and synaptogenesis in the developing brain (Lombardo et al., 1995; Benson and Salton, 1996; Snyder et al., 1998b). Mouse VGF knockouts (KO) are small, hypermetabolic, and have reduced leptin levels, suggesting a prominent role in energy metabolism (Hahm et al., 1999). These findings provide evidence for a neurotrophic role for VGF, in addition to its function as neuroendocrine molecule.

The cAMP responsive event modulator (CREM) gene encodes both antagonists and activators of the cAMP-dependent transcriptional response by alternative splicing (for review see Della Fazia et al., 1997). CREM is inducible by activation of the cAMP signaling pathway with the kinetics of an early response gene. An alternatively splice repressor form, inducible cAMP early repressor (ICER), may be important for the transient nature of cAMP-induced gene expression. The role of this molecule in regenerative signaling remains to be determined.

We found two genes, ATF3 and GAP43, whose expression differed markedly between cAMP treatment and conditioning lesion. These differences suggest possible differences in mechanism between these pro-regenerative paradigms. ATF3 is part of the activating transcription factor/CREB family of transcription factors. The gene encodes a leucine zipper transcription factor (Hsu et al., 1991) that is increased by cellular stress (reviewed in Hai et al., 1999). ATF3 is strongly induced by sciatic nerve lesion in dorsal root ganglia and spinal motor neurons (Tsujino et al., 2000) and in the geniculate ganglion after chorda tympani injury (Tsuzuki et al., 2002). ATF3 represses transcription as a homodimer (Chen et al., 1994) and activates transcription as a heterodimer with cJun (Hai and Curran, 1991; Chu et al., 1994). Jun has been investigated as a RAG (Broude et al., 1997; Houle et al., 1998; Lerch et al., 2014), and the possibility of cJun and ATF3 acting as inducers of RAG expression has been validated in DRG (Seijffers et al., 2007). Whether ATF3 might be acting as a transcriptional activator or repressor in these models must be investigated further.

Surprisingly, GAP43 expression decreased in our cAMP model (Figure 1). GAP43 is the prototypical RAG and is often used as a marker for regenerating axons (Benowitz et al., 1990). Schreyer and colleagues have previously shown that GAP43 may be repressed by cAMP (Andersen et al., 2000a,b). In a search for the signal that causes DRG neurons to downregulate GAP43 after they reach their targets, this group found decreases on both the mRNA and protein levels in cAMP-exposed DRG neurons. Interestingly, exposure of rat cortical neurons to spinal cord extract, but not extract from cerebellum or muscle, was sufficient to decrease GAP43, and this repression was adenyl cyclase-dependent (Karimi-Abdolrezaee and Schreyer, 2002). The finding of increased neurite outgrowth associated with GAP43 repression challenged the widespread notion that regeneration is accompanied by GAP43 induction. This has subsequently been shown in other models of regeneration, such as following cJun activation of axon growth (Lerch et al., 2014).

## Implications

These studies produced three surprising results. First, we found little overlap in the gene expression changes produced by the three regeneration paradigms we studied. Although the neurons in each of these models all express high levels of cAMP in the regenerative state, the changes in transcription differ markedly. Rather than uncovering a core regeneration program that allows a switch from inhibition to growth in the CNS environment, we found different transcriptional changes, each leading to a similar regenerative phenotype. This suggests the existence of several transcriptional programs with overlapping function.

Second, we found that the first three transcripts studied are each sufficient to elicit regeneration *in vivo*. In addition, both SLPI and MT are necessary for the regeneration induced by cAMP or conditioning lesion. This remarkable “hit rate” could be serendipity, or it could tell us that regeneration may not require a coordinated program requiring multiple gene products. It could be that IL-6, SLPI, and MT all create a coordinated cellular response of several pathways. But it is heartening that regeneration can be produced with each of these varied approaches as monotherapy. Whether the effects of these therapies in combination would be more effective is a critical question for future study. In addition, in the animal studies done so far, no loss of function has been done in CNS resident neurons. But these experiments are more difficult since CNS neurons do not regeneration spontaneously, thus requiring the use of a regeneration competent background (e.g., PTEN deletion, as has been done for DLK with the optic nerve injury model; (Watkins et al., 2013). Finally, the function of the regenerated axons has not yet been tested by measures of physiology or behavior.

Finally, the gene for GAP43, critical to regeneration after conditioning lesion, is strongly downregulated by cAMP treatment. This evidence argues strongly that these different regenerative paradigms act through diverse transcriptional programs. Rather than a single path to successful regeneration, many possible transcriptional changes may lead to re-growth. Mapping these paths will be critical to flipping the transcriptional switch from inhibition to growth in the CNS.

## Conflict of Interest Statement

The authors declare that the research was conducted in the absence of any commercial or financial relationships that could be construed as a potential conflict of interest.
